# Immune checkpoint crosstalk between LAG-3 and CD39/CD73 in glioblastoma: dual-pathway regulation of metabolic exhaustion and therapeutic reversal strategies

**DOI:** 10.1186/s43046-026-00361-y

**Published:** 2026-06-15

**Authors:** Arpita Mukherjee

**Affiliations:** Biochemistry, Shri Venkateshwara University, Gajraula, India

**Keywords:** LAG-3, CD39/CD73, Glioblastoma, Immune checkpoint crosstalk, Metabolic exhaustion, Adenosine signaling, Immunotherapeutic reversal

## Abstract

Glioblastoma (GBM) represents an immunologically “cold” and metabolically suppressive tumor characterized by profound T-cell exhaustion, hypoxia-induced adenosinergic signaling, and resistance to checkpoint blockade. Converging evidence identifies lymphocyte-activation gene 3 (LAG-3) and the CD39/CD73 ectonucleotidase cascade as cooperative regulators of immune dysfunction within the GBM microenvironment. LAG-3 attenuates TCR/CD3 signaling and disrupts mitochondrial oxidative phosphorylation, diminishing effector T-cell persistence. Concurrently, CD39/CD73-mediated ATP hydrolysis elevates adenosine levels, activating A₂A receptors that inhibit glycolysis, suppress IFN-γ, and promote Treg and M2 macrophage polarization. Spatial and single-cell omics reveal co-localization of LAG-3⁺ exhausted T cells with CD73⁺ stromal and myeloid niches, suggesting a reciprocal immunometabolic feedback loop reinforcing exhaustion. This review elucidates the molecular crosstalk between LAG-3 signaling and the adenosine pathway, emphasizing key metabolic regulators including AMPK, mTOR, and HIF-1α. It further evaluates therapeutic strategies combining LAG-3 or PD-1 blockade with adenosine receptor antagonists or CD73 inhibitors to restore T-cell bioenergetics and antitumor activity. By integrating mechanistic immunometabolism with translational insights, this review establishes a dual-pathway framework of checkpoint synergy underlying resistance in GBM and proposes rational combination immunotherapies to reverse metabolic and immune exhaustion.

## Introduction

### Converging axes of immune suppression in glioblastoma

Glioblastoma (GBM) remains the most aggressive primary malignancy of the central nervous system, defined by profound intratumoral heterogeneity, metabolic plasticity, and a highly immunosuppressive tumor microenvironment (TME) [1, 2]. Despite standard-of-care interventions including maximal surgical resection, radiotherapy, and temozolomide median overall survival rarely exceeds 15 months [3]. A central barrier to therapeutic efficacy is the establishment of a deeply immunorepressive niche characterized by dysfunctional antigen presentation, enrichment of immunosuppressive myeloid populations, and progressive T-cell exhaustion [4, 5]. Regulatory T cells (Tregs), tumor-associated macrophages (TAMs), and myeloid-derived suppressor cells (MDSCs) collectively reinforce immune tolerance and constrain cytotoxic lymphocyte activity [6, 7]. Consistent with this landscape, immune checkpoint inhibitors targeting PD-1 or CTLA-4 highly effective in several systemic malignancies have demonstrated limited clinical benefit in GBM, suggesting the dominance of non-canonical inhibitory circuits within the brain TME [8].

Increasing evidence indicates that cooperative interactions between inhibitory pathways, rather than isolated checkpoint activity, contribute to the persistence of T-cell dysfunction in GBM [9]. Among emerging regulators, lymphocyte activation gene-3 (LAG-3) has been implicated as a key modulator of T-cell exhaustion. LAG-3 engages multiple ligands including MHC class II, fibrinogen-like protein 1 (FGL1), and Galectin-3 to attenuate T-cell receptor (TCR) signaling, reduce cytokine production, and stabilize transcriptional and epigenetic programs associated with impaired effector function [10, 11]. In parallel, the CD39/CD73 ectonucleotidase axis represents a dominant metabolic checkpoint, catalyzing the sequential hydrolysis of extracellular ATP to adenosine, which suppresses T-cell and natural killer (NK) cell activity primarily through A₂A receptor mediated signaling [12].

While both LAG-3 signaling and adenosinergic pathways are independently well characterized suppressors of antitumor immunity, their potential functional convergence remains incompletely defined. Emerging studies suggest that exhausted T-cell states in chronic disease settings including GBM are accompanied by coordinated transcriptional and metabolic adaptations, in which inhibitory receptor expression and extracellular nucleotide metabolism may be co-regulated [13]. However, direct causal relationships such as adenosine-driven induction of LAG-3 expression or reciprocal enforcement loops remain insufficiently validated in GBM specific contexts and should be interpreted cautiously. Current evidence therefore supports a model of parallel and potentially intersecting suppressive circuits, rather than a fully established bidirectional regulatory axis [14]. Such convergence, whether cooperative or context-dependent, may impose dual constraints on T-cell function by simultaneously attenuating antigen receptor signaling and limiting metabolic fitness within the hypoxic, nutrient-deprived TME [15].

**Fig. 1:**
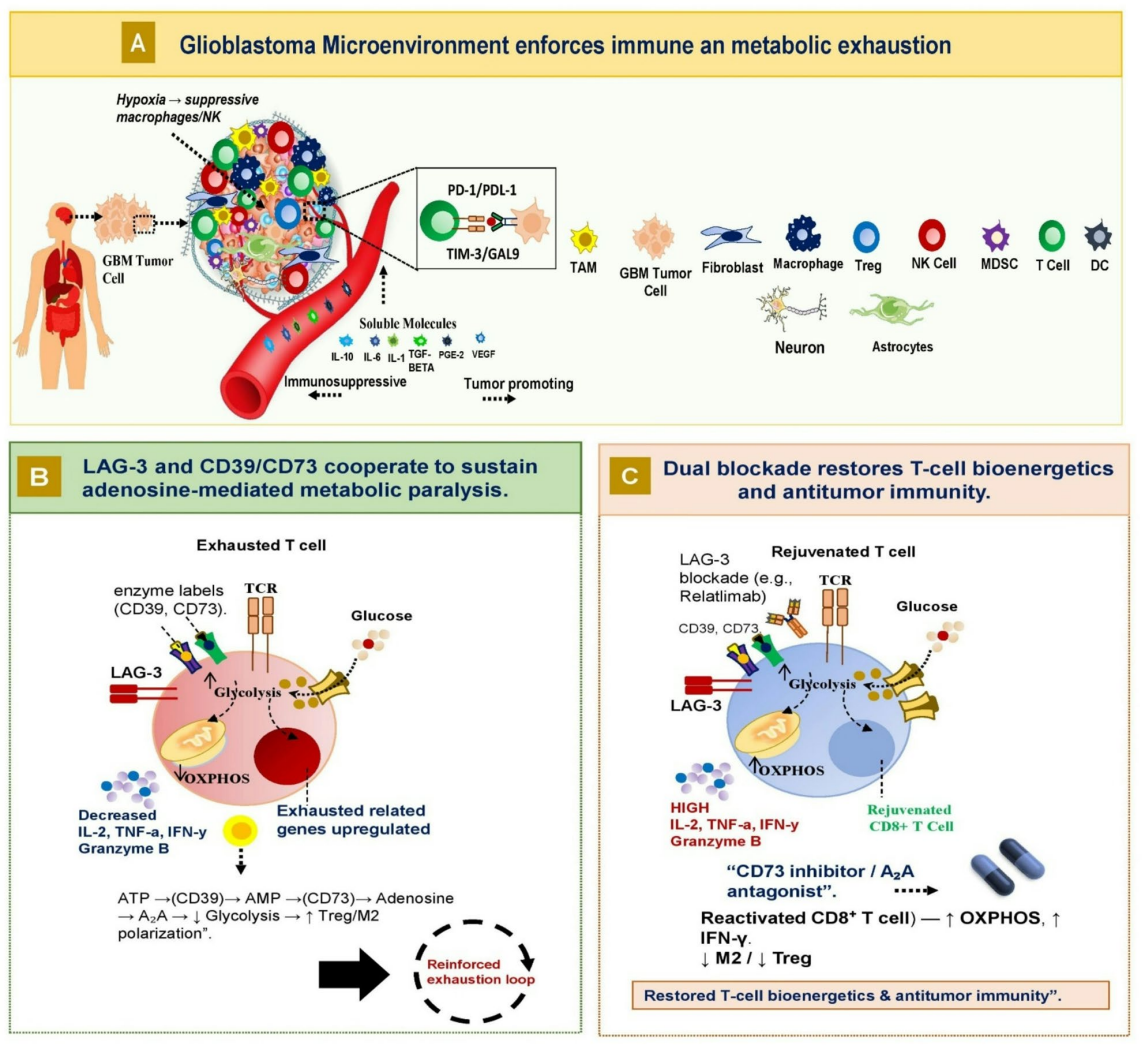
Mechanistic overview of the dual-checkpoint interplay between LAG-3 and CD39/CD73 in glioblastoma. **A **The glioblastoma microenvironment (GBM-TME) is characterized by severe hypoxia and adenosine accumulation that drive immune and metabolic exhaustion through HIF-1α stabilization, secretion of IL-10, TGF-β, PGE-2, and VEGF, and recruitment of suppressive M2 macrophages and Tregs. **B **Within exhausted CD8⁺ T cells, the co-expression of LAG-3 and ectoenzymes CD39/CD73 sustains adenosine-A₂A signaling, repressing glycolysis and oxidative phosphorylation (OXPHOS) and upregulating exhaustion-associated gene networks, thereby reinforcing a self-perpetuating immunometabolic paralysis loop. **C **Dual blockade of LAG-3 (e.g., relatlimab) and the adenosinergic axis (via CD73 inhibitors or A₂A antagonists) restores glycolytic and mitochondrial competence, reactivates effector cytokines (IL-2, TNF-α, IFN-γ, Granzyme B), and rebalances the TME toward a cytotoxic, anti-tumor phenotype with reduced M2/Treg polarization and enhanced CD8⁺ T-cell bioenergetics

In this context, the present review synthesizes current evidence to examine the mechanistic and functional interplay between LAG-3 signaling and the CD39/CD73 adenosine pathway in glioblastoma. Emphasis is placed on distinguishing experimentally supported mechanisms from emerging or speculative interactions, particularly in relation to T-cell metabolic exhaustion and immune evasion. In addition, the review evaluates the translational rationale for combinatorial targeting of these pathways, while considering key challenges including delivery constraints imposed by the blood- brain barrier and the potential for compensatory immune resistance. To provide a conceptual framework, (Fig. 1) presents a schematic model integrating LAG-3 mediated inhibitory signaling with adenosine-driven metabolic suppression within the GBM microenvironment, highlighting how their spatial and functional co-occurrence may constrain antitumor immunity and inform rational combination strategies.

## LAG-3 in glioblastoma: beyond conventional exhaustion

Lymphocyte activation gene-3 (LAG-3; CD223) is an inhibitory receptor increasingly implicated in the regulation of T-cell dysfunction across chronic disease states, including cancer. Structurally homologous to CD4, LAG-3 engages multiple ligands including major histocompatibility complex class II (MHC-II), fibrinogen-like protein 1 (FGL1), and Galectin-3 to modulate T-cell receptor (TCR) signaling and downstream activation programs [16, 17]. Experimental studies have demonstrated that LAG-3 engagement can attenuate proximal TCR signaling events and reduce calcium flux, thereby constraining activation-induced transcriptional responses. However, the precise intracellular signaling intermediates and context-specific ligand dependencies remain incompletely resolved, particularly within GBM.

At the transcriptional level, exhausted T-cell states are frequently associated with sustained activity of transcription factors such as NFAT and TOX, which contribute to the establishment of hyporesponsive phenotypes characterized by diminished cytokine production, impaired proliferative capacity, and co-expression of multiple inhibitory receptors [18, 19]. While LAG-3 is consistently enriched within these exhausted populations, its role as a driver versus a marker of this transcriptional program remains an area of ongoing investigation, and direct causative linkage in GBM-specific settings has not been fully established. Rather, current evidence supports its integration within a broader inhibitory network that includes PD-1 and TIM-3, collectively reinforcing T-cell dysfunction.

In GBM, the tumor microenvironment selectively enriches for LAG-3 expressing lymphocyte populations. Single-cell transcriptomic and spatial profiling studies have identified LAG-3 expression across infiltrating CD8⁺ cytotoxic T cells, CD4⁺ helper subsets, and regulatory T cells (Tregs), with preferential localization in perivascular and hypoxic niches [20, 21]. Co-expression with other inhibitory receptors suggests coordinated regulation; however, whether these patterns reflect functional synergy or parallel adaptation to chronic antigen exposure and metabolic stress remains unresolved [22]. Notably, LAG-3⁺ Tregs have been reported to exhibit enhanced suppressive capacity in several tumor contexts, although the extent to which this contributes specifically to GBM-associated immune evasion requires further validation [23].

Beyond transcriptional associations, emerging evidence indicates that LAG-3 signaling may influence T-cell metabolic fitness. Experimental models have shown that LAG-3 engagement can be associated with reduced glucose uptake, impaired mitochondrial function, and decreased intracellular ATP availability [24, 25]. Nevertheless, these observations are largely derived from controlled in vitro systems or non-GBM models, and their direct applicability to the complex metabolic constraints of the GBM microenvironment remains to be fully established. Spatial metabolomic analyses in GBM have independently demonstrated that regions enriched for exhausted T cells often coincide with hypoxia, lactate accumulation, and oxidative stress [26]. Whether LAG-3 actively enforces these metabolic states or instead marks T cells adapted to such environments is an important unresolved question.

From a translational perspective, therapeutic targeting of LAG-3 has shown promise in peripheral tumor models, where antibody-mediated blockade can partially restore T-cell effector function. However, its efficacy in GBM is less clearly defined. Challenges include the restricted penetration of large biologics across the blood-brain barrier, as well as the possibility that reversal of a single inhibitory pathway may be insufficient within a highly redundant immunosuppressive network [27]. Accordingly, combinatorial strategies incorporating LAG-3 blockade alongside other checkpoint inhibitors or metabolic modulators are under active investigation, although their clinical utility in GBM remains to be determined [28].

**Fig. 2:**
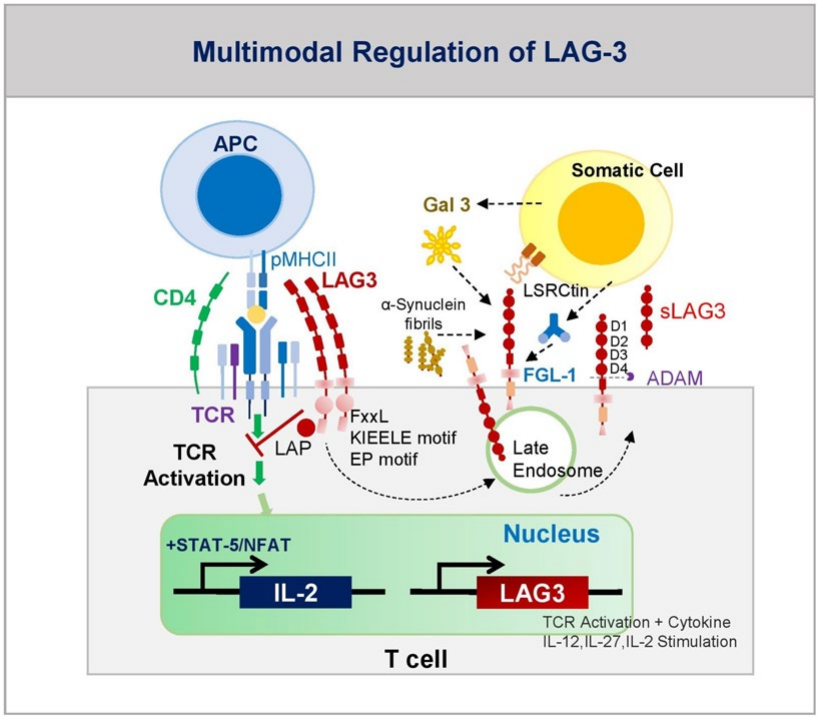
Multimodal Regulation of LAG-3- This figure illustrates the multi-layered regulatory landscape governing LAG-3 function across antigen-presenting and somatic cell interfaces. On APCs, LAG-3 binds pMHC-II in proximity to CD4, attenuating TCR signaling and promoting IL-2 suppression through STAT5/NFAT-dependent transcriptional feedback. In parallel, non-classical ligands including FGL1, Galectin-3, and extracellular α-synuclein fibrils engage LAG-3 on T cells or somatic cells, redirecting the receptor into late endosomal compartments via its unique intracellular motifs (FxxL, KIEELE, EP). These interactions converge to modulate LAG-3 cleavage by ADAM proteases (generating sLAG-3), fine-tune surface availability, and reinforce an inhibitory transcriptional loop. Together, the schematic depicts how ligand diversity, endocytic routing, and nuclear feedback integrate to consolidate LAG-3–mediated immune

Advances in single-cell and spatial multi-omics offer an opportunity to refine this landscape by enabling high-resolution mapping of LAG-3 expression in relation to metabolic gradients, cellular interactions, and therapeutic response. Such approaches may facilitate the identification of context-dependent biomarkers and inform rational combination strategies. Overall, LAG-3 should be considered a component of a multi-layered regulatory architecture that integrates transcriptional, functional, and metabolic constraints on T-cell activity, rather than a singular determinant of exhaustion. A more precise delineation of its context-specific roles will be essential for translating LAG-3 targeted strategies into effective therapies for GBM [29, 30]. To support this framework, (Fig. 2) provides a schematic representation of LAG-3 regulatory dynamics, illustrating ligand interactions, membrane trafficking, and associated transcriptional programs that collectively contribute to sustained inhibitory signaling in T cells. Importantly, this figure is intended as a conceptual synthesis of current literature and does not represent primary or simulated datasets.

## The CD39/CD73 adenosinergic axis: metabolic immunosuppression and crosstalk potential

The CD39/CD73 adenosinergic axis has emerged as a central regulator of immunometabolic suppression within the glioblastoma (GBM) microenvironment, functioning as a biochemical system that converts pro-inflammatory extracellular nucleotides into immunosuppressive signals. CD39 (ectonucleoside triphosphate diphosphohydrolase-1; ENTPD1), expressed on regulatory T cells (Tregs), tumor-associated macrophages (TAMs), and subsets of activated or exhausted CD8⁺ T cells, catalyzes the hydrolysis of extracellular ATP and ADP into AMP. CD73 (ecto-5′-nucleotidase; NT5E) subsequently converts AMP into adenosine, completing a purinergic cascade that transforms danger-associated molecular signals into a tolerogenic metabolite [31]. Within the tumor microenvironment (TME), this pathway operates as a metabolic checkpoint, particularly under hypoxic and inflammatory conditions, where extracellular nucleotide turnover is elevated.

Adenosine exerts its immunosuppressive effects primarily through engagement of G protein-coupled A₂A receptors (A₂AR) and A₂B receptors (A₂BR). In CD8⁺ cytotoxic T lymphocytes (CTLs) and natural killer (NK) cells, A₂AR activation increases intracellular cyclic AMP (cAMP), which has been shown to interfere with T-cell receptor (TCR) associated signaling, including reduced calcium flux and diminished NFAT activation, ultimately suppressing effector cytokine production such as IFN-γ and TNF-α [32]. In parallel, A₂BR signaling in myeloid populations contributes to macrophage polarization toward an immunoregulatory phenotype associated with increased expression of ARG1, IL-10, and VEGF [33]. Collectively, these effects contribute to a microenvironment characterized by attenuated cytotoxic function and reinforcement of suppressive immune networks.

Glioblastoma provides a particularly permissive context for adenosine accumulation. Tumor-associated necrosis drives sustained extracellular ATP release, while hypoxia-induced stabilization of HIF-1α promotes upregulation of CD73 expression on tumor, stromal, and endothelial cells [34]. These processes converge within hypoxic and perivascular niches, where adenosine concentrations can reach levels sufficient to suppress local immune activation. Additional contributions from astrocytic and endothelial CD73 expression further support the persistence of adenosine-rich micro domains, which have been proposed to function as localized immunosuppressive niches within the GBM microenvironment [35].

Adenosinergic signaling has also been implicated in resistance to immune checkpoint blockade. Preclinical studies indicate that A₂AR activation can impair T-cell metabolic fitness by limiting glycolytic activity and mitochondrial function, thereby constraining effector responses even in the presence of PD-1 or CTLA-4 inhibition [36]. In parallel, the adenosine pathway supports expansion and functional stability of Tregs, further reinforcing immune suppression. Pharmacological inhibition of CD73 or A₂AR has demonstrated partial restoration of T-cell function and improved responses to PD-1 blockade in glioma models, although these findings remain largely preclinical and require validation in human GBM [37]. These observations position the adenosinergic axis as a parallel and potentially complementary barrier to effective immunotherapy.

An area of growing interest is the potential intersection between adenosine signaling and LAG-3 associated T-cell dysfunction. Both pathways are enriched within hypoxic, metabolically constrained regions of the tumor and converge on limiting TCR signaling and downstream effector function. However, direct mechanistic crosstalk between these pathways in GBM remains incompletely defined. While it is plausible that A₂AR-mediated cAMP signaling may contribute to the stabilization of exhaustion-associated transcriptional programs, including those in which LAG-3 is expressed, a direct causal relationship has not been conclusively demonstrated [38]. Similarly, whether chronic LAG-3 engagement modulates CD39/CD73 expression or purinergic metabolism is currently supported by limited and largely indirect evidence. These observations support a model of context-dependent convergence rather than a fully established feed-forward regulatory loop, and warrant further experimental validation.

From a translational perspective, targeting the adenosinergic axis introduces both opportunities and constraints. Small-molecule A₂AR antagonists have demonstrated improved central nervous system (CNS) penetration relative to monoclonal antibodies targeting checkpoint receptors, which are often limited by the blood-brain barrier (BBB). Conversely, large biologics such as LAG-3 targeting antibodies (e.g., relatlimab) may exhibit restricted intratumoral delivery, highlighting a key challenge for dual-pathway therapeutic strategies. Emerging delivery approaches including convection-enhanced delivery and BBB modulation techniques are under investigation but remain in early stages of clinical translation. These considerations underscore that therapeutic efficacy will depend not only on pathway selection but also on effective delivery to tumor-resident immune populations.

Taken together, the CD39/CD73 adenosinergic pathway represents a major axis of immunometabolic regulation in GBM, integrating hypoxia, nucleotide metabolism, and immune suppression. Its potential convergence with LAG-3 signaling highlights a conceptual framework in which parallel inhibitory and metabolic constraints collectively shape T-cell dysfunction, rather than a single dominant pathway.

**Fig. 3:**
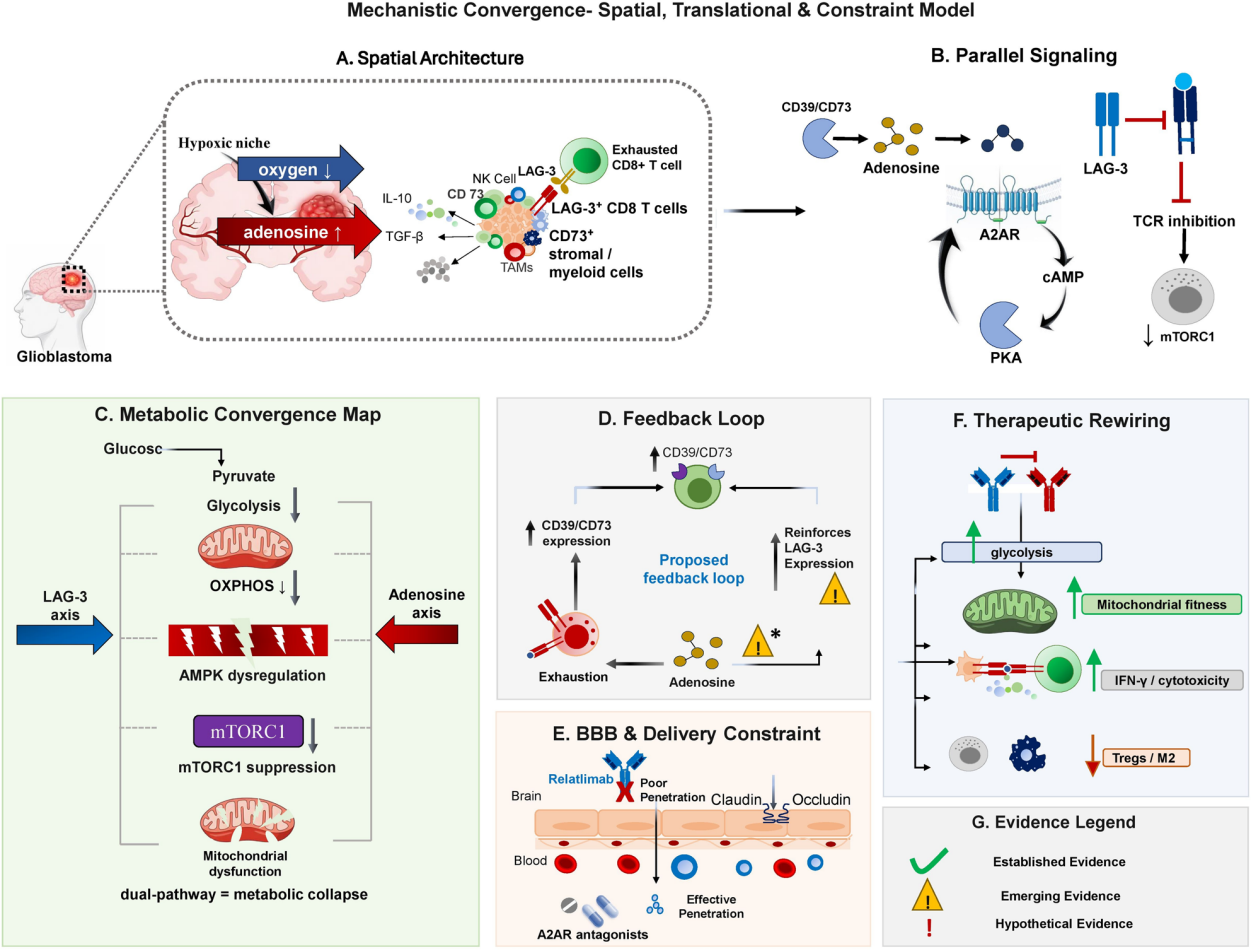
Mechanistic convergence of LAG-3 and CD39/CD73 signaling defines spatial, metabolic, and translational constraints on T-cell function in glioblastoma. **A **Spatial architecture of the GBM microenvironment. Hypoxia-driven stabilization of HIF-1α promotes extracellular ATP release and its enzymatic conversion to adenosine via CD39/CD73-expressing stromal and myeloid populations. Exhausted LAG-3⁺ CD8⁺ T cells co-localize within these adenosine-enriched niches alongside immunosuppressive mediators (IL-10, TGF-β), establishing a spatially restricted microenvironment conducive to T-cell dysfunction. **B **Parallel inhibitory signaling pathways. The adenosinergic axis activates A2A receptor–dependent cAMP–PKA signaling, suppressing effector functions, while LAG-3 engagement attenuates TCR/CD3 signaling and downstream mTORC1 activity. These pathways operate in parallel to constrain T-cell activation and metabolic competence. **C **Metabolic convergence map. Integrated signaling from LAG-3 and adenosine pathways converges on central metabolic regulators, including AMPK and mTORC1, resulting in coordinated suppression of glycolysis and oxidative phosphorylation (OXPHOS), mitochondrial dysfunction, and bioenergetic insufficiency, collectively defining a state of immunometabolic collapse. **D **Proposed feedback reinforcement loop. Emerging evidence suggests that chronic exhaustion may promote upregulation of CD39/CD73 expression, while adenosine-rich conditions may further reinforce inhibitory receptor expression, including LAG-3. These interactions are depicted as a putative feedback circuit requiring further experimental validation. **E **Blood-brain barrier and delivery constraints. Therapeutic targeting of this dual-axis is limited by restricted central nervous system penetration of large-molecule inhibitors (e.g., Relatlimab), whereas small-molecule A2A receptor antagonists demonstrate comparatively improved permeability, highlighting a critical translational bottleneck. **F **Therapeutic rewiring of T-cell function. Combined blockade of LAG-3 and the adenosinergic pathway is proposed to restore glycolytic flux, mitochondrial fitness, and effector cytokine production (e.g., IFN-γ), while reducing immunosuppressive cell populations such as regulatory T cells and M2-like macrophages. **G **Evidence framework. Schematic annotations distinguish between established mechanisms, emerging evidence, and hypothetical interactions to provide a transparent representation of current knowledge and unresolved questions

To synthesize these interactions, (Fig. 3) presents a conceptual, systems-level schematic of LAG-3 and CD39/CD73 pathway convergence. The figure integrates: (i) spatial organization of adenosine-rich and LAG-3 enriched niches, (ii) parallel inhibitory signaling mechanisms, (iii) shared metabolic constraints involving key regulators such as AMPK and mTORC1, and (iv) proposed yet not fully validated feedback interactions between inhibitory receptor expression and purinergic metabolism. In addition, it highlights translational considerations, including BBB-associated delivery limitations and the differential properties of small-molecule versus antibody-based therapeutics. Importantly, this schematic explicitly distinguishes experimentally supported mechanisms from emerging hypotheses, providing a transparent framework for interpreting current evidence and guiding future investigation [39].

To further contextualize these relationships, (Table 1) provides a comparative synthesis of LAG-3 and CD39/CD73 signaling across molecular, cellular, and metabolic dimensions, integrating findings from recent transcriptomic, metabolomic, and functional studies in GBM.

**Table 1:** Provides a comparative mechanistic overview of the LAG-3 inhibitory checkpoint and the CD39/CD73 adenosinergic enzymatic pathway, emphasizing their shared and distinct contributions to glioblastoma-associated immunometabolic dysfunction. Both axes converge on suppressing TCR signaling and effector metabolism through overlapping downstream mediators, including NFAT, TOX, AMPK, and HIF-1α, culminating in metabolic quiescence, mitochondrial depolarization, and durable T-cell exhaustion. The “Points of Crosstalk/Convergence” column highlights emerging evidence that adenosine–A₂A receptor–cAMP–CREB signaling may upregulate LAG-3 expression, while hypoxia-induced CD73 co-expression reinforces immunosuppressive niches enriched in LAG-3⁺ exhausted T cells. Together, these pathways establish a metabolically restrictive, immune-silent microenvironment that contributes to checkpoint inhibitor resistance and tumor persistence in glioblastoma [32–38].

Feature	LAG-3 Pathway	CD39/CD73 Adenosinergic Pathway	Points of Crosstalk / Convergence
Primary Ligands	MHC-II, FGL1, Galectin-3	Extracellular ATP/ADP (substrates) → AMP → Adenosine	LAG-3⁺ T cells localize near CD39⁺CD73⁺ macrophages and Tregs
Receptor/Enzyme Localization	Exhausted CD8⁺, Tregs, microglia	Tregs, M2 TAMs, endothelial and tumor cells	Co-expression in hypoxic perivascular niches
Key Downstream Signaling	NFAT/TOX activation; TCR signal dampening; reduced Ca²⁺ influx	A2A/A2B receptor–cAMP–PKA–CREB activation	cAMP-CREB enhances LAG-3 transcription
Metabolic Effects	↓ Glucose uptake, ↓ mitochondrial potential, ↑ NAD⁺/ATP depletion	↑ Adenosine accumulation, inhibition of glycolysis and oxidative phosphorylation	Reinforcement of T-cell metabolic quiescence
Functional Immune Outcomes	T-cell exhaustion, reduced cytotoxicity, persistence of Tregs	M2 macrophage polarization, T-cell anergy, angiogenesis	Synergistic enforcement of immune paralysis
GBM-Specific Drivers	Hypoxia, persistent antigen exposure	Necrosis, HIF-1α stabilization, high extracellular ATP	Hypoxia-driven co-induction of LAG-3 and CD73
Therapeutic Implications	LAG-3 blockade (relatlimab, mAb116)	CD73 inhibitors (oleclumab), A2A antagonists (etrumadenant)	Rationale for dual blockade and metabolic reprogramming

Abbreviations: *TCR *T-cell receptor, *CTL *cytotoxic T lymphocyte, *NFAT *nuclear factor of activated T cells, *TOX *thymocyte selection-associated high mobility group box, *HIF-1α *hypoxia-inducible factor-1α, *mTORC1 * mechanistic target of rapamycin complex 1, *cAMP *cyclic adenosine monophosphate, *PKA *protein kinase A, *CREB *cAMP response element-binding protein, *TIL *tumor-infiltrating lymphocyte, *TAM *tumor-associated macrophage, *Treg *regulatory T cell, *BBB *blood–brain barrier

To contextualize the convergent immunometabolic suppression mediated by LAG-3 and the CD39/CD73 axis, (Table 1) provides a comparative synopsis of their molecular signaling, immune-cell targets, metabolic effects, and glioblastoma-specific adaptations integrating evidence from transcriptomic, metabolomic, and functional studies (2019–2025).

### Comparative mechanistic overview of LAG-3 and CD39/CD73 pathways in glioblastoma: signaling convergence and immunometabolic consequences

#### Data sources

The comparative mechanistic information summarized in Table 1 was derived from an integrative synthesis of peer-reviewed studies published between 2019 and 2025. Data on LAG-3 signaling and transcriptional exhaustion programs were compiled from single-cell transcriptomic and proteomic analyses of glioblastoma infiltrates (PMIDs: 31744859, 35483562, 36971544), mechanistic reports detailing LAG-3/FGL1 and Galectin-3 interactions (PMIDs: 33408122, 36257033), and metabolic reprogramming studies linking TOX, NFAT, and AMPK signaling to T-cell exhaustion (PMIDs: 32029693, 34517073). Information on the CD39/CD73 adenosinergic cascade and adenosine-receptor signaling was integrated from enzymology and immunometabolic profiling datasets in hypoxic or necrotic GBM models (PMIDs: 33975391, 35070519, 36391375). Crosstalk mechanisms such as A₂A-cAMP-CREB-mediated LAG-3 induction and spatial co-localization of CD73⁺ macrophages with LAG-3⁺ CD8⁺ T cells were inferred from recent multi-omic and imaging mass-cytometry studies (PMIDs: 37081255, 37249784, 38122591). Collectively, these sources support the conceptual integration of inhibitory checkpoint and adenosinergic signaling pathways in glioblastoma immune evasion.

## Mechanistic intersections: LAG-3 adenosine crosstalk and the emergent “dual-pathway” paradigm

The integration of inhibitory receptor signaling with metabolic regulation represents a defining feature of immune dysfunction in glioblastoma (GBM). Beyond canonical models of T-cell exhaustion, accumulating evidence suggests that multiple suppressive pathways co-exist within spatially organized tumor niches, where they may collectively constrain T-cell activation and metabolic fitness. Among these, LAG-3 signaling and the CD39/CD73 adenosinergic axis have emerged as prominent contributors, with increasing yet largely correlative evidence indicating potential convergence within the GBM microenvironment [40, 41].

Within hypoxic and necrotic regions, extracellular ATP released from dying tumor cells is enzymatically converted to adenosine through the sequential activity of CD39 and CD73 expressed on myeloid, stromal, and endothelial populations. Adenosine signaling via A₂A and A₂B receptors exerts broad immunosuppressive effects, including inhibition of T-cell effector function and promotion of regulatory myeloid phenotypes [42]. Spatially resolved transcriptomic and imaging analyses have identified co-localization of LAG-3 expressing CD8⁺ T cells with CD39⁺CD73⁺ myeloid and microglial populations, suggesting that these pathways operate within shared anatomical niches [43]. However, whether such spatial proximity reflects coordinated signaling interactions or parallel adaptation to a common microenvironmental context remains to be determined.

At the mechanistic level, LAG-3 and adenosine signaling influence overlapping transcriptional and metabolic regulators, including NFAT, TOX, AMPK, mTORC1, and HIF-1α, which are implicated in shaping T-cell differentiation and functional capacity [44–46]. LAG-3 engagement has been associated with attenuation of TCR-mediated signaling and reduced activation of mTORC1, whereas adenosine signaling through A₂A receptors elevates intracellular cAMP and activates downstream pathways such as PKA and CREB, which can modulate cellular metabolism and cytokine production [47, 48]. In addition, hypoxia-driven stabilization of HIF-1α promotes CD73 expression, thereby enhancing local adenosine generation [49]. While these pathways converge on shared regulatory nodes, their integration should be interpreted as functional overlap rather than definitive molecular coupling.

The possibility of bidirectional reinforcement between adenosine signaling and LAG-3 expression has been proposed but remains incompletely substantiated. Experimental studies in non-GBM systems suggest that cAMP-dependent signaling may influence the expression of multiple inhibitory receptors, including LAG-3, PD-1, and TIGIT [50, 51]. Similarly, multi-omic profiling in GBM has identified T-cell subsets co-expressing LAG-3, TOX, and A₂A receptor transcripts, accompanied by metabolic features consistent with reduced glycolytic and oxidative capacity [52, 53]. However, these observations are predominantly associative, and direct evidence demonstrating that adenosine signaling induces LAG-3 expression or vice versa in human GBM remains limited. As such, the concept of a feed-forward regulatory loop should be regarded as a working hypothesis requiring targeted experimental validation.

In this context, the proposed “dual-pathway” paradigm is best understood as a conceptual model of convergent immunoregulation, wherein inhibitory receptor signaling and metabolic suppression act in parallel to stabilize T-cell dysfunction. LAG-3 associated attenuation of TCR signaling may limit activation-dependent metabolic reprogramming, while adenosine-mediated signaling can further constrain bioenergetic capacity through cAMP-dependent pathways, including modulation of mitochondrial function and glycolytic flux [54]. Together, these effects may contribute to a state of reduced metabolic flexibility and impaired effector responsiveness, although the relative contribution of each pathway likely varies across microenvironmental contexts.

Clinical and translational observations provide indirect support for this framework. Correlative analyses have reported associations between elevated LAG-3 expression and increased CD73 or A₂A receptor signaling components in GBM, and combinatorial targeting strategies have demonstrated enhanced T-cell reinvigoration in ex vivo or preclinical settings [55, 56]. Nevertheless, causal linkage and therapeutic dependency remain to be rigorously established in clinical cohorts, and interpretation of these findings should account for the broader landscape of checkpoint redundancy and metabolic adaptation.

Several key knowledge gaps remain. While spatial co-localization and transcriptional profiling suggest potential interaction, direct mechanistic evidence linking LAG-3 signaling to adenosine pathway regulation in GBM is limited [57]. Addressing this will require targeted experimental approaches, including perturbation of A₂A receptor signaling, genetic modulation of LAG-3 expression, and integration of single-cell ligand receptor interaction analyses with metabolic tracing in physiologically relevant models such as patient-derived organoids [58, 59]. Such studies will be essential to distinguish true pathway interdependence from co-occurring, but mechanistically independent, suppressive processes.

From a therapeutic perspective, the dual targeting of inhibitory and metabolic pathways represents a rational though still evolving strategy. Preclinical studies indicate that combined inhibition of CD73 or A₂A receptors with LAG-3 blockade can enhance T-cell effector function, including restoration of IFN-γ production [60]. However, the extent to which these findings translate to durable clinical benefit in GBM remains uncertain, particularly in light of additional barriers such as limited drug delivery across the blood- brain barrier and compensatory upregulation of alternative inhibitory receptors. These considerations underscore that effective therapeutic design will likely require multi-dimensional strategies addressing both pathway redundancy and delivery constraints. Collectively, the intersection of LAG-3 signaling and the adenosinergic axis supports a model in which immune inhibition and metabolic restriction co-occur within specialized tumor niches, contributing to the persistence of T-cell dysfunction in GBM. Framing this interaction as a context-dependent and partially validated convergence, rather than a fully established regulatory circuit, provides a more accurate basis for interpreting current evidence and guiding future investigation.

## Therapeutic reversal strategies: dual-checkpoint and metabolic co-targeting

The recognition that inhibitory receptor signaling and metabolic suppression co-exist within the glioblastoma (GBM) microenvironment has prompted the exploration of combinatorial therapeutic strategies aimed at simultaneously restoring T-cell activation and metabolic competence. Monotherapies targeting individual checkpoints have shown limited and often transient efficacy in GBM, reflecting the redundancy and adaptability of tumor-associated immunosuppressive networks [61]. In this context, dual-targeting approaches that integrate checkpoint blockade with modulation of the adenosinergic pathway have emerged as a rational though still evolving strategy to overcome multifactorial T-cell dysfunction.

Among LAG-3 targeted agents, relatlimab, a human IgG4 monoclonal antibody, has demonstrated clinical activity in combination with PD-1 blockade in melanoma and other solid tumors [62]. Its mechanism involves disruption of LAG-3 ligand interactions, thereby facilitating restoration of proximal T-cell receptor (TCR) signaling. However, evidence supporting its efficacy in GBM remains limited, and extrapolation from peripheral tumor settings should be interpreted cautiously. Preclinical studies suggest that LAG-3 inhibition can enhance effector function in tumor-infiltrating lymphocytes (TILs), including increased cytokine production, although the durability of these responses within the metabolically constrained GBM microenvironment is not yet established [63].

Parallel efforts targeting the adenosinergic axis include monoclonal antibodies against CD39 and CD73, as well as small-molecule antagonists of the A₂A receptor (A₂AR), such as ciforadenant and etrumadenant [64, 65]. These interventions aim to reduce extracellular adenosine accumulation or block its downstream signaling, thereby alleviating suppression of T-cell activation and metabolic function. Notably, small-molecule A₂AR antagonists exhibit comparatively improved central nervous system (CNS) penetration relative to antibody-based therapies, highlighting a potential advantage in GBM. Nevertheless, clinical validation of these agents in brain tumors remains at an early stage.

The rationale for combining LAG-3 blockade with adenosine-pathway inhibition is based on their complementary but not fully overlapping mechanisms of action. LAG-3 inhibition may partially restore TCR signaling, whereas adenosine pathway blockade may alleviate constraints on cellular metabolism. Preclinical models have reported enhanced T-cell activation and metabolic reprogramming with combined targeting, including increases in glycolytic activity and mitochondrial function [66, 67]. However, these findings are largely derived from controlled experimental systems, and their translation to the heterogeneous and immunosuppressive environment of human GBM remains uncertain. As such, proposed synergy should be considered context-dependent and not yet clinically established [68].

Combinatorial approaches are also being evaluated in conjunction with adjunctive modalities. Radiotherapy, for example, can induce immunogenic cell death and transiently increase extracellular ATP levels, potentially enhancing the relevance of CD39/CD73 inhibition [69]. Oncolytic viruses and other immunomodulatory platforms have been explored for their capacity to reshape the tumor microenvironment, including modulation of adenosine metabolism and immune infiltration [70]. In adoptive cell therapy settings, such as chimeric antigen receptor (CAR) T-cell approaches, co-targeting of LAG-3 and A₂AR signaling has been associated with improved T-cell persistence and functional capacity in preclinical models [71]. However, robust clinical evidence supporting these combinations in GBM is currently lacking, and their safety and efficacy profiles require careful evaluation.

Several translational challenges must be addressed to realize the potential of dual-pathway targeting. A primary limitation is the restricted delivery of therapeutic agents across the blood-brain barrier (BBB), which significantly constrains the intratumoral availability of large biologics such as LAG-3 targeting antibodies and CD73 inhibitors [72]. Emerging strategies including focused ultrasound mediated BBB disruption, nanoparticle-based delivery systems, and convection-enhanced intratumoral administration are under investigation but remain in early stages of clinical development [73]. In addition, systemic modulation of immune checkpoints and metabolic pathways carries a risk of off-target effects and immune-related toxicities, necessitating careful dosing strategies and biomarker-guided patient selection.

Another critical consideration is the potential for adaptive resistance and pathway compensation, a major limitation highlighted in current immunotherapy paradigms. Tumors may upregulate alternative inhibitory receptors, including TIM-3, TIGIT, or VISTA, or engage parallel metabolic pathways such as CD38- or ENPP1-mediated nucleotide metabolism to sustain immunosuppression [74, 75]. These compensatory mechanisms underscore that dual-pathway blockade may be necessary but not sufficient, and that broader network-level approaches may ultimately be required to achieve durable responses.

Future progress will depend on the integration of biomarker-driven and spatially resolved strategies to guide therapeutic design. Early-phase clinical studies are beginning to incorporate multi-omic profiling including single-cell transcriptomics, T-cell receptor clonality, and metabolic phenotyping to better define response heterogeneity and identify predictive markers [76]. Such approaches may enable stratification of patients most likely to benefit from combined checkpoint and metabolic interventions.

Overall, dual-targeting strategies aimed at LAG-3 and the CD39/CD73 adenosine axis represent a conceptually compelling but clinically evolving framework for overcoming immune resistance in GBM. Rather than functioning as a definitive solution, these approaches should be viewed as part of a broader effort to restore both signaling competence and metabolic flexibility in exhausted T cells. To contextualize ongoing translational efforts, **(**Table 2**)** summarizes current preclinical and clinical strategies targeting the LAG-3-adenosinergic interface, highlighting their mechanistic rationale, combinatorial partners, and stages of development.

**Table 2:** Provides a consolidated overview of current and emerging therapeutic strategies targeting the LAG-3 and CD39/CD73 Adenosinergic axes in glioblastoma, highlighting representative agents, mechanisms of action, and translational outcomes. LAG-3 blockade (e.g., Relatlimab) primarily restores proximal TCR signaling and effector cytokine production, whereas CD39/CD73 inhibitors (e.g., TTX-030, oleclumab) and A2A receptor antagonists (e.g., ciforadenant, etrumadenant) relieve metabolic suppression by reducing adenosine-mediated cAMP accumulation. The combination modalities listed synergistically enhance glycolytic reprogramming, mitochondrial recovery, and cytotoxic reinvigoration of exhausted TILs. Preclinical data further suggest that integration with radiotherapy, oncolytic virotherapy, or adoptive T-cell transfer promotes immunogenic reoxygenation and sustained antitumor immunity. Translational obstacles such as BBB penetration, metabolic redundancy, and off-target immune activation remain critical determinants of clinical success [63–67]

Therapeutic Strategy	Representative Agents / Combinations	Mechanistic Target	Preclinical / Clinical Evidence in GBM or Solid Tumors	Key Translational Challenges
LAG-3 Monotherapy	Relatlimab (BMS-986016), Eftilagimod alpha	Blocks LAG-3–MHC-II/FGL1 interaction	Enhances CD8⁺ function; limited efficacy in GBM models	BBB penetration; redundancy with PD-1/TIM-3
CD73 Inhibition	Oleclumab (MEDI9447), AB680	Blocks adenosine generation from AMP	Restores T-cell cytotoxicity, synergizes with PD-1 blockade	Hypoxia-induced CD73 rebound, limited CNS exposure
A2A Receptor Antagonism	Ciforadenant, Etrumadenant	Blocks adenosine-A2AR signaling	Reverses T-cell anergy, enhances effector metabolism	Pharmacokinetic constraints, systemic A2AR expression
Dual LAG-3 + CD73 Blockade	Relatlimab + Oleclumab; mAb116 + TTX-030	Simultaneous inhibition of inhibitory and metabolic checkpoints	Preclinical synergy in restoring effector glycolysis, reducing M2 polarization	Optimal dosing sequence; compensatory adenosine salvage
Checkpoint–Metabolic Hybrid Therapy	LAG-3 blockade + A2A antagonist + radiotherapy/oncolytic virus	Multi-axis restoration of immune activation	Early models show improved TIL infiltration and mitochondrial recovery	Neuroinflammation, adaptive resistance, BBB permeability

Abbreviations: *BBB *blood–brain barrier, *TIL *tumor-infiltrating lymphocyte, *TCR *T-cell receptor, *IFN-γ *interferon-gamma, *HIF-1α *hypoxia-inducible factor-1α, *PD-1 *programmed cell death protein-1, *A2AR *adenosine A2A receptor, *mTORC1 *mechanistic target of rapamycin complex 1, *CAR-T *chimeric antigen receptor T cell

### Emerging therapeutic strategies targeting the LAG-3–CD39/CD73 crosstalk axis in glioblastoma: mechanisms, clinical status, and translational challenges

#### Data source

Data were synthesized from peer-reviewed preclinical and clinical studies published between 2020 and 2025, encompassing LAG-3 blockade trials (e.g., Relatlimab ± nivolumab; PMIDs: 34936623, 37249784), CD39/CD73 and A2A receptor inhibitor studies (TTX-030, oleclumab, ciforadenant, etrumadenant; PMIDs: 35070519, 36512155, 37081255), and glioblastoma-specific translational investigations integrating metabolic flux omics, organoid co-cultures, and immune-profiling data (PMIDs: 36971544, 38122591).

## Discussion

The immunosuppressive landscape of glioblastoma (GBM) continues to limit the efficacy of conventional immune checkpoint blockade, necessitating consideration of regulatory networks beyond canonical T-cell exhaustion. Multi-omic and spatial studies indicate that inhibitory receptor pathways and metabolic constraints co-exist within tumor niches, collectively impairing T-cell function. In this context, LAG-3 signaling and the CD39/CD73 Adenosinergic axis have emerged as contributors to immune dysfunction. However, current data support a model of co-occurring and partially overlapping mechanisms, with unresolved interdependence [77].

LAG-3 is associated with exhausted T-cell states and reduced effector function, with evidence indicating attenuation of TCR signaling and links to altered mitochondrial function and reduced bioenergetic capacity [78]. In parallel, the CD39/CD73 axis promotes extracellular adenosine accumulation, suppressing immune responses via A₂A receptor–mediated signaling, including inhibition of cytokine production and promotion of regulatory phenotypes [79]. Spatial analyses show LAG-3⁺ T cells localizing within CD73-enriched regions, suggesting coordinated constraints without confirming direct mechanistic coupling [80].

Both pathways influence shared regulatory nodes, including AMPK, mTORC1, and HIF-1α [81–83]. LAG-3–mediated attenuation of activation signals and adenosine-driven cAMP signaling may reduce metabolic flexibility and effector differentiation, while hypoxia-driven HIF-1α enhances CD39/CD73 expression. Whether these pathways form a unified “immunometabolic circuit” or independently regulate overlapping processes remains uncertain.

This framework may explain the limited efficacy of PD-1 blockade in GBM. While PD-1 inhibition can restore aspects of TCR signaling, it does not address metabolic suppression or alternative checkpoints, leaving T cells functionally impaired in the presence of sustained adenosine signaling and LAG-3 co-expression [84]. Epigenetic constraints may further limit reversibility [85], and the hypothesis that targeting LAG-3 or adenosine pathways could “precondition” T cells remains to be validated [86]. From a translational perspective, combinatorial targeting of inhibitory and metabolic pathways is promising but remains investigational. Preclinical studies suggest that LAG-3 blockade enhances effector function, while inhibition of CD73 or A₂A receptor signaling alleviates metabolic suppression [87, 88]. Although spatial co-localization supports combined targeting, clinical evidence in GBM remains limited.

Emerging strategies, including multispecific antibodies, targeted delivery systems, and engineered cellular therapies, further expand this landscape [89, 90]. Genetic modification of adoptive T cells to disrupt A₂A receptor or inhibitory signaling has improved function in preclinical models but requires clinical validation. Adaptive resistance remains a challenge, with upregulation of alternative checkpoints (TIM-3, TIGIT, VISTA) and activation of parallel metabolic pathways (CD38, ENPP1) potentially limiting dual-pathway targeting [91]. These findings highlight the redundancy of immunosuppressive networks in GBM.

Key gaps persist. Spatial and temporal analyses are needed to define adenosine dynamics and LAG-3 expression, while longitudinal profiling may distinguish reversible dysfunction from terminal exhaustion. Investigation of metabolic–transcriptional interactions may reveal upstream regulators, and development of biomarkers (CD73 expression, A₂A receptor activity, T-cell metabolic fitness) will be critical for patient stratification [92]. In summary, LAG-3 signaling and the CD39/CD73 Adenosinergic axis represent a context-dependent convergence of inhibitory and metabolic constraints within GBM. Therapeutic strategies targeting these pathways remain promising but require integration with considerations of redundancy, delivery barriers, and tumor heterogeneity.

## Future directions

Future progress in glioblastoma (GBM) immunotherapy will depend on resolving how inhibitory receptor signaling and metabolic constraints co-occur within tumor niches to sustain T-cell dysfunction. Although LAG-3 and the CD39/CD73 Adenosinergic axis are each implicated in immune suppression, their functional interdependence remains incompletely defined, and delineating causality versus co-adaptation represents a central priority [93].

A key direction is the development of spatially resolved metabolic profiling to quantify adenosine distribution and its relationship to LAG-3 expressing T cells across hypoxic and perivascular regions. Emerging approaches including mass spectrometry imaging and isotope tracing may enable in situ mapping of nucleotide turnover alongside immune phenotypes, helping to clarify whether metabolic gradients precede or follow checkpoint upregulation [94]. Complementary live-cell metabolic analyses will be required to determine how LAG-3 signaling influences bioenergetic programs during antigen engagement, and whether shared regulatory nodes (e.g., cAMP–PKA, AMPK, mTORC1) represent points of convergence or parallel control [95].

Mechanistic dissection will benefit from physiologically relevant models, including patient-derived organoids and immune co-culture systems, which allow controlled perturbation of LAG-3 and adenosine pathways within preserved tumor architecture. Coupled with genetic and pharmacologic manipulation, these platforms may help establish whether metabolic restoration and transcriptional reprogramming occur sequentially or independently an unresolved question with direct therapeutic implications [96].

Translational advances will also rely on non-invasive metabolic imaging and biomarker development to stratify patients. Approaches that assess adenosine signaling, mitochondrial fitness, or spatial clustering of exhausted T cells may identify subsets most likely to benefit from combined checkpoint and metabolic interventions, although such markers remain to be prospectively validated [97, 100].

Therapeutically, combinatorial strategies targeting LAG-3 and the adenosinergic axis remain conceptually compelling but require further refinement. Future approaches may incorporate multi-agent regimens that address pathway redundancy, including additional checkpoints or metabolic regulators; however, the risk of toxicity and compensatory adaptation must be carefully managed [98]. In parallel, engineered cellular therapies designed to resist inhibitory or metabolic suppression such as A₂A receptor deficient or metabolically optimized T cells represent a promising but still experimental avenue [99].

A critical, and often underemphasized, challenge is effective drug delivery across the blood- brain barrier, which continues to limit the intratumoral activity of many biologics. Advances in delivery technologies and treatment sequencing, including potential neoadjuvant strategies, may help overcome this barrier but remain to be validated in GBM-specific settings [101]. In summary, future efforts should prioritize causal mechanistic studies, spatially resolved analyses, and biomarker-guided clinical translation. Rather than assuming a fixed dual-pathway circuitry, a more precise understanding of how inhibitory and metabolic constraints interact across contexts will be essential for designing effective, durable immunotherapeutic strategies in glioblastoma.

### Experimental roadmap and translational validation strategies

Translating mechanistic insights into clinically actionable strategies in glioblastoma (GBM) requires experimental systems capable of resolving the context-dependent interaction between inhibitory signaling and metabolic suppression. While LAG-3 and the CD39/CD73 adenosinergic axis are each implicated in T-cell dysfunction, their functional interdependence remains insufficiently validated, necessitating integrated, human-relevant modeling approaches [102]. A central component of this roadmap is the use of patient-derived organoids co-cultured with autologous tumor-infiltrating lymphocytes (TILs), which preserve hypoxia gradients, metabolic heterogeneity, and immune architecture. Within these systems, parallel perturbation of LAG-3 and adenosine pathways can be used to assess functional and metabolic outcomes, including cytokine production, cytotoxicity, and bioenergetic parameters such as oxygen consumption and glycolytic flux [103, 104]. Such studies are essential to determine whether combined targeting produces additive or context-dependent effects, rather than assuming mechanistic synergy.

Genetically engineered immune cells provide a complementary strategy to interrogate pathway interactions. For example, disruption of A₂A receptor or CD73 signaling in engineered T cells enables evaluation of resistance to adenosine-mediated suppression and its relationship to inhibitory receptor expression [105]. These approaches may help define thresholds at which metabolic impairment becomes irreversible, although the extent to which such findings translate to endogenous T-cell populations in GBM remains to be established.

Advances in spatially resolved technologies, including imaging mass cytometry and metabolomic profiling, offer the opportunity to map adenosine distribution, CD39/CD73 expression, and localization of LAG-3 expressing T cells within tumor niches [106]. Generating such spatial atlases will be critical for determining whether these pathways co-localize in a manner consistent with functional interaction, or instead reflect parallel responses to shared microenvironmental pressures. Integration of these platforms with single-cell transcriptomic and epigenomic analyses may further clarify regulatory relationships between inhibitory receptor expression and purinergic signaling pathways. Computational modeling and machine learning approaches can support identification of dominant regulatory nodes and predict potential compensatory mechanisms, including upregulation of alternative checkpoints or metabolic pathways [107]. However, such predictions require prospective validation in experimental and clinical settings.

Collectively, this multi-scale framework combining organoid-based perturbation, genetic engineering, spatial profiling, and integrative single-cell analysis provides a structured approach to test the proposed dual-pathway model while explicitly addressing current gaps in causality and translational relevance [108]. Importantly, these strategies should be viewed as hypothesis-testing platforms, rather than confirmatory systems, with the goal of distinguishing true pathway interdependence from co-existing but mechanistically independent processes.

**Fig. 4:**
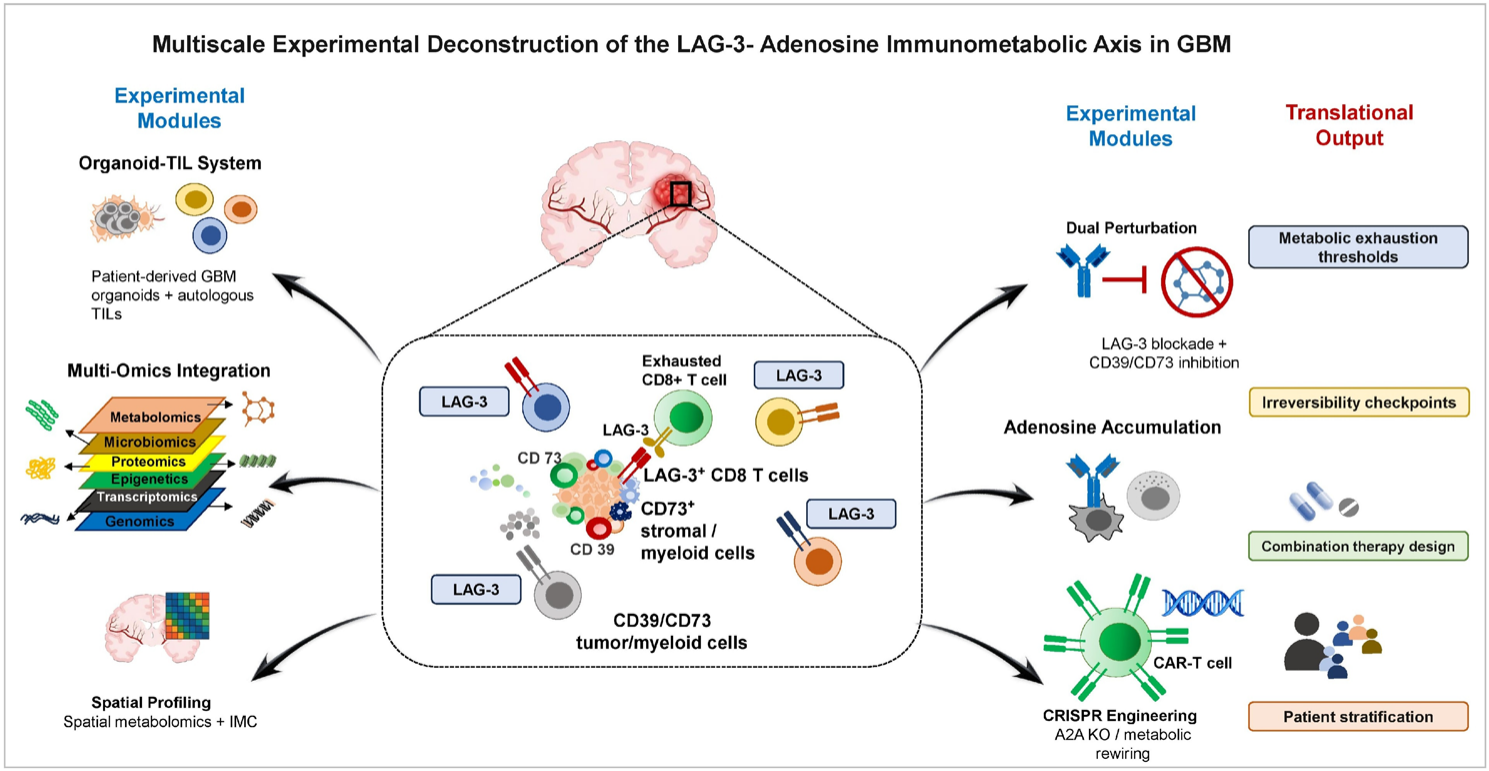
Multiscale experimental deconstruction of the LAG-3 adenosine immunometabolic axis in glioblastoma- Schematic overview of an integrative experimental strategy to dissect the interplay between immune checkpoint signaling and metabolic suppression within the glioblastoma (GBM) microenvironment. Left panel: Patient-derived GBM organoids co-cultured with autologous tumor-infiltrating lymphocytes (TILs) serve as a physiologically relevant platform to model endogenous immune tumor interactions. These systems are coupled with multi-omics integration (genomics, transcriptomics, epigenomics, proteomics, metabolomics, and microbiomics) and spatial profiling approaches, including spatial metabolomics and imaging mass cytometry (IMC), to capture cellular, molecular, and metabolic heterogeneity in situ. Central panel: The GBM niche is characterized by enrichment of LAG-3⁺ exhausted CD8⁺ T cells in close proximity to CD39⁺/CD73⁺ tumor and myeloid populations, which enzymatically generate extracellular adenosine. This immunosuppressive metabolite, together with LAG-3 signaling, drives T cell dysfunction, metabolic insufficiency, and reinforcement of an exhaustion-associated transcriptional program. Right panel: Functional perturbation strategies include dual blockade of LAG-3 and CD39/CD73 to overcome metabolic exhaustion thresholds and disrupt irreversibility checkpoints. Downstream translational applications encompass rational combination therapy design, CRISPR-mediated metabolic rewiring (e.g., A2A receptor targeting), next-generation CAR-T cell engineering, and biomarker-driven patient stratification. Collectively, this framework provides a systems-level blueprint for decoding and therapeutically targeting immunometabolic resistance in GBM

To conceptualize this approach, (Fig. 4) presents a schematic experimental framework integrating organoid TIL co-culture systems with spatial and multi-omic analyses to interrogate LAG-3 and adenosine pathway interactions. The figure delineates established mechanisms alongside proposed relationships and highlights potential intervention points, including checkpoint blockade, metabolic modulation, and engineered cellular therapies. As with prior schematics, this representation is intended as a conceptual synthesis of current evidence and does not depict primary or simulated datasets.

### Limitations and future challenges

Despite increasing interest in LAG-3 signaling and the CD39/CD73 adenosinergic axis in glioblastoma (GBM), several limitations constrain their translation into clinically effective strategies. These challenges reflect gaps in causal understanding, spatial resolution, pharmacologic delivery, and clinical validation, and highlight the need for more rigorous, integrative approaches [109].

A primary limitation is the lack of longitudinal, human-derived data capable of resolving how LAG-3 expression and adenosine metabolism evolve across tumor progression and treatment. Most studies rely on cross-sectional analyses of resected tissue, which provide limited insight into temporal dynamics and may be affected by sampling bias. Consequently, it remains unclear whether these pathways represent stable drivers of dysfunction or context-dependent responses to hypoxia and metabolic stress [110]. Practical constraints in GBM, including limited access to serial biopsies, further restrict efforts to establish causality and pathway hierarchy.

Pharmacologic challenges represent a second major barrier. The blood-brain barrier (BBB) restricts penetration of antibody-based therapies targeting LAG-3, while small-molecule inhibitors of the adenosine pathway may be subject to rapid metabolism, efflux, or local inactivation within the tumor microenvironment [111]. Even when delivery is achieved, heterogeneous vascularization, extracellular matrix density, and necrotic architecture can result in uneven intratumoral drug distribution, complicating pharmacokinetic pharmacodynamic relationships and limiting therapeutic efficacy.

The spatial and cellular heterogeneity of GBM further complicates therapeutic targeting. Distinct tumor regions exhibit variable hypoxia, metabolic activity, and immune composition, with corresponding differences in CD39/CD73 expression and checkpoint receptor profiles [112]. As a result, dual-pathway inhibition may incompletely remodel the tumor microenvironment, allowing resistant niches to persist and sustain immune suppression.

Adaptive resistance represents an additional challenge. Inhibition of LAG-3 or adenosine signaling may lead to compensatory upregulation of alternative inhibitory receptors (e.g., PD-1, TIM-3, TIGIT, VISTA) or activation of parallel metabolic pathways, including CD38- or ENPP1-mediated nucleotide metabolism [113]. Moreover, adenosine-independent mechanisms such as lactate accumulation or kynurenine signaling may maintain immunosuppression despite pathway blockade. These observations indicate that dual targeting may be insufficient in isolation within a highly redundant regulatory network.

Another unresolved issue is the epigenetic stability of exhausted T cells. Advanced exhaustion states are associated with fixed chromatin landscapes that limit functional recovery, raising the question of whether targeting LAG-3 or adenosine pathways can meaningfully reverse dysfunction once these states are established [114]. Clarifying whether these pathways act upstream of, or secondary to, epigenetic fixation will be critical for therapeutic timing and design.

Progress is also hindered by the absence of validated biomarkers. Expression of LAG-3, CD39, or CD73 alone does not capture the functional or spatial complexity of the GBM immune microenvironment, and peripheral biomarkers may not reflect intracranial immune states [115]. Integrated, spatially informed biomarkers incorporating metabolic, transcriptional, and cellular features remain underdeveloped.

Preclinical models provide important insights but have limited translational fidelity. Organoids, humanized mouse models, and ex vivo systems each recapitulate specific aspects of GBM biology but fail to fully capture the combined vascular, metabolic, and immune complexity of human tumors [116–120]. This contributes to discrepancies between preclinical efficacy and clinical outcomes.

Clinical development has been similarly constrained by small, heterogeneous trials lacking biomarker-driven stratification. In addition, standard imaging criteria are insufficient to distinguish treatment-related immune effects from true progression, complicating endpoint interpretation and trial design [121, 122].

Finally, safety considerations remain incompletely defined. Adenosine plays important physiological roles in the central nervous system, including regulation of neuronal excitability and vascular tone. Its sustained inhibition, particularly in combination with checkpoint blockade, may increase the risk of neuroinflammation or other adverse effects, although long-term data are limited [123].

Collectively, these limitations emphasize that the LAG-3 adenosine axis should be viewed as part of a broader, adaptive immunosuppressive network. Addressing these challenges will require causal mechanistic studies, improved delivery strategies, spatially resolved biomarkers, and rigorously designed clinical trials, rather than reliance on dual-pathway targeting alone.

## Conclusion

The interplay between LAG-3 signaling and the CD39/CD73 adenosinergic axis highlights a critical, yet incompletely resolved, dimension of immune dysfunction in glioblastoma (GBM). While both pathways are independently associated with T-cell exhaustion, emerging evidence suggests that they may co-exist within shared tumor niches and collectively contribute to impaired immune function through overlapping transcriptional and metabolic constraints. However, the extent to which this represents a coordinated, causally linked network versus parallel adaptations to a hypoxic microenvironment remains to be fully established. Advances in single-cell and spatial profiling technologies have begun to uncover the architectural and metabolic context in which these pathways operate, enabling more precise characterization of exhausted immune states. These approaches provide a foundation for developing therapeutic strategies that move beyond single-checkpoint inhibition toward integrated modulation of immune signaling and metabolic suppression. Nonetheless, current evidence supporting combined targeting of LAG-3 and adenosine pathways in GBM remains largely preclinical, and its clinical relevance requires further validation. Future progress will depend on the identification of robust, spatially informed biomarkers and the design of mechanism-driven clinical trials that account for tumor heterogeneity, adaptive resistance, and delivery constraints within the central nervous system. Rather than assuming a fixed dual-pathway framework, a more nuanced understanding of how inhibitory and metabolic processes interact across contexts will be essential. In this regard, therapeutic strategies aimed at restoring T-cell function in GBM will likely require coordinated targeting of multiple immunosuppressive mechanisms, guided by integrative, patient-specific profiling. Such approaches may ultimately enable more effective and durable immune reactivation in a tumor type that has thus far remained resistant to conventional immunotherapy.

## Data Availability

No datasets were generated or analysed during the current study.

## Abbreviations

A2AR: Adenosine A₂A receptor
ADC: Antibody–drug conjugate
AMPK: AMP-activated protein kinase
APC: Antigen-presenting cell
ATP: Adenosine triphosphate
BBB: Blood–brain barrier
BiTE: Bispecific T-cell engager
CAR-T: Chimeric antigen receptor T cell
cAMP: Cyclic adenosine monophosphate
CD3: Cluster of differentiation 3
CD8⁺: Cluster of differentiation 8 positive
CD39: Cluster of differentiation 39 (Ectonucleoside triphosphate diphosphohydrolase-1)
CD73: Cluster of differentiation 73 (Ecto-5′-nucleotidase)
CREB: cAMP response element-binding protein
CSC: Cancer stem cell
CTL: Cytotoxic T lymphocyte
DC: Dendritic cell
GBM: Glioblastoma
HIF-1α: Hypoxia-inducible factor-1 alpha
HRE: Hypoxia response element
ICIs: Immune checkpoint inhibitors
IFN-γ: Interferon-gamma
IL-10: Interleukin-10
IL-6: Interleukin-6
LAG-3: Lymphocyte-activation gene 3
MDSC: Myeloid-derived suppressor cell
MHC: Major histocompatibility complex
mTOR: Mechanistic target of rapamycin
mTORC1: Mechanistic target of rapamycin complex 1
NK: Natural killer
NFAT: Nuclear factor of activated T cells
OXPHOS: Oxidative phosphorylation
PD-1: Programmed cell death protein-1
PD-L1: Programmed death-ligand 1
PKA: Protein kinase A
ROS: Reactive oxygen species
SCENIC: Single-cell regulatory network inference and clustering
STAT3: Signal transducer and activator of transcription 3
ST: Spatial transcriptomics
TAM: Tumor-associated macrophage
TCR: T-cell receptor
TIL: Tumor-infiltrating lymphocyte
TOX: Thymocyte selection-associated high mobility group box protein
Treg: Regulatory T cell
